# In-hive learning of specific mimic odours as a tool to enhance honey bee foraging and pollination activities in pear and apple crops

**DOI:** 10.1038/s41598-022-22985-5

**Published:** 2022-11-28

**Authors:** Walter M. Farina, Andrés Arenas, Paula C. Díaz, Cinthia Susic Martin, María J. Corriale

**Affiliations:** 1 Laboratorio de Insectos Sociales, Departamento de Biodiversidad y Biología Experimental, Facultad de Ciencias Exactas y Naturales, Buenos Aires, Argentina; 2 Instituto de Fisiología, Biología Molecular y Neurociencias (IFIBYNE), CONICET-Universidad de Buenos Aires, Buenos Aires, Argentina; 3 Grupo de Estudios sobre Biodiversidad en Agroecosistemas, Departamento de Ecología, Genética y Evolución, Facultad de Ciencias Exactas y Naturales, Buenos Aires, Argentina; 4 Instituto de Ecología, Genética y Evolución de Buenos Aires (IEGEBA), CONICET-Universidad de Buenos Aires, Buenos Aires, Argentina; 5 Present Address: Instituto Nacional de Medicina Tropical, Administración Nacional de Laboratorios e Institutos de Salud (ANLIS), Ministerio de Salud de la Nación, Puerto Iguazú, Misiones Argentina

**Keywords:** Ecology, Zoology

## Abstract

The areas devoted to agriculture that depend on pollinators have been sharply increased in the last decades with a concomitant growing global demand for pollination services. This forces to consider new strategies in pollinators’ management to improve their efficiency. To promote a precision pollination towards a specific crop, we developed two simple synthetic odorant mixtures that honey bees generalized with their respective natural floral scents of the crop. We chose two commercial crops for fruit production that often coexist in agricultural settings, the apple (*Malus domesticus*) and the pear trees (*Pyrus communis*). Feeding colonies with sucrose solution scented with the apple mimic (AM) or the pear mimic (PM) odour enabled the establishment of olfactory memories that can bias bees towards the flowers of these trees. Encompassing different experimental approaches, our results support the offering of scented food to improve foraging and pollination activities of honey bees. The circulation of AM-scented sucrose solution inside the hive promoted higher colony activity, probably associated with greater activity of nectar foragers. The offering of PM-scented sucrose solution did not increase colony activity but led to greater pollen collection, which is consistent with pear flowers offering mainly pollen as resources for the bees. Results obtained from apple and pear crops suggest that the offering of AM- and PM-scented sucrose solution increased fruit yields. This preliminary study highlights the role of in-hive olfactory learning to bias foraging preferences within pome fruit crops.

## Introduction

With growing global populations, sustaining and indeed increasing food production in a way that does not compromise the environment is the major and burning challenge of our times^1^. The production of approximately 70% of the leading single crops, accounting for up to 35% of global food production, increases with animal pollination, that is, when animals, mainly invertebrates, transfer pollen from male to female flowers^2^. Due to the steady decline in the abundance and diversity of wild pollinators caused by intensive use of agrochemicals, landscape fragmentation, habitat destruction and pollution^3^, insect pollination service strongly relies on managed species such as the honey bee *Apis mellifera*^4,5^. Yet, the global stock of honey bee colonies does not meet current crop pollination demands^1,6,7^. Such circumstances have led to consider new strategies for the management of pollinators to improve their efficiency in agroecosystems. Among the most economically important crops that depend on bee-mediated pollination are the pome fruit trees, represented mainly by apple and pear trees. Pollination of some varieties, such as the ‘Red Delicious’ apple or ‘Packham’ pear, increases the quality of fruit, but also the quantity by 20–30% and even more depending on the cultivar^9–12^.

Pollination is a mutualism in which the plant receives the insects’ pollination service in return for a reward. Pollinators might select flowers based on their innate preferences to certain floral cues, but also on learned preferences. Like many other insects, honey bee foragers associate neutral cues such as floral odours with the rewards (i.e., nectar or pollen) the flower provides^13^. In turn, odour-reward associations lead to memories that improve foraging efficiency by guiding the bees towards the learned stimulus^15–18^. As a central place forager, the honey bee can learn floral odours not only when visiting rewarding flowers but also inside the nest, for instance, when scented nectars collected by foragers is unloaded and distributed among nestmates^20–21^. Such social learning is key for adaptive collective responses, as it enables hive mates to acquire information about different foraging options directly inside the nest. The retrieval of olfactory information acquired in the social context strongly promotes the search for new foraging sites^13,22^.

Olfactory information transfer is also functional when odours are not introduced by foragers, but directly provided inside the nest by scenting the food of the hive^23,24^. During the 1940s and 1950s, attempts to increase honey and seed yields by guiding bees through the offering of food scented with the fragrance of the crop flower showed ambiguous results^25^. More recently, it has been showed that feeding colonies food scented with a simple synthetic odorant mixture that mimics sunflower scent increased foraging activity and recruitment towards the sunflower whilst produced significant gains in seed yields^26^.

Unlike sunflower monocultures, crops grown in more heterogeneous environments pose a greater challenge to direct foraging bee visits. In environments where several crops bloom massively and overlapping, pollination services may be negatively affected when bees switch between different options according to changes in their availability and/or type of resource they provide, or according to colony needs^28–29^. To what extent olfactory memory established by the offering of scented food within the honey bee hive are stable enough to bias the preferences of their foragers in a diverse and fluctuating environment is still unknown.

In the Rio Negro valley, south of Argentina, pear (*Pyrus communis*) and apple (*Malus domesticus*) crops commonly occur together and can compete for pollination services as they exhibit successive and overlapping flowering events^10,31–32^. Pear and apple trees, which do not usually exceed a dozen hectares, can be grown on the same plot (rows of plants of one species interspersed with rows of plants of the other) or in neighbouring plots within the foraging range of the same bees. Both crops diverge substantially in the amount of pollen and nectar their flowers provide, with pear flowers offering mainly pollen and apple flowers offering mainly nectar^31,32^. Bees visiting pear blossoms often switch to more rewarding plants when protein demands of the nest decrease, resulting in insufficient pollination and low pear production. The risk to switch to alternative flowers might take also place in apple crops since it is often co-flowers with different attractive crops^29^. This scenario therefore provides an opportunity to investigate whether olfactory memories established by the offering of scented foods improve pollination efficiency of mass flowering crops that compete for the services honey bee colonies provide. To this end, we develop specific mimic odours for the pear and the apple flowers that we used to scent the sugar solution we offered inside the hives. Then we compared the number of incoming bees in hives fed with sugar solution scented or not with the mimics, as an indicator of colony activity since most of these bees are expected to return from foraging sites^33^. In addition, we measured the amount of collected pollen in both groups of colonies, and estimated the fruit sets for the apple and the pear plots they pollinated.

## Results

### Generalization of memories from pear mimic odours to natural floral scents

**Figure 1:**
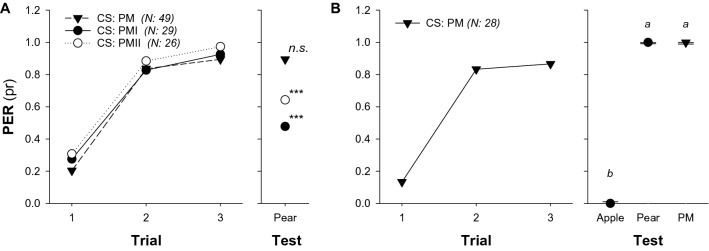
Generalization of memories from pear mimic odours to natural floral scents. (**A**) Generalization was tested towards the single presentation of the pear natural odour (right panel) after one of the pear mimics (PM, PMI or PMII) was used as conditioned stimulus (CS) during an absolute classical conditioning of the proboscis extension reflex (PER; left panel). Asterisks indicate significant differences between responses obtained at the third conditioning trial and the test (***, p < 0.001). No significant difference (n.s.) indicates that bees could not discriminate between PM (3rd trial) and the unrewarded pear natural scent (PER-test). (**B**) Generalization was evaluated towards the single presentation of the natural apple and pear odour (right panel) after PM (from **A**) was used as CS during the absolute PER conditioning (left panel). Same letter indicates no significant differences between the generalized response of pear natural odour and the response retrieved by the PM itself. The experimental subjects were all foraging bees completely naïve for the CS that had no access to any pear tree. Symbols indicate the proportion of PER and bars (in test) show the 95% confidence intervals. Numbers between brackets indicate sample size. Package ‘lme4’, version 1.1-30. https://github.com/lme4/lme4/.

We developed three different pear flower mimics (PM, PMI or PMII) and investigated to what extent their conditioned responses, acquired in absolute proboscis extension reflex (PER) conditioning assays, generalize to the pear natural floral scent. For all three mixtures tested, the so-called PM showed the highest level of generalization (PM vs. Pear: Z = − 1.336, P = 0.1815; PMI vs. Pear: Z = − 3.104, P = 0.0019; PMII vs. Pear: Z = − 2.628, P = 0.0086; Fig. 1A), indicating that bees perceived it similarly to the pear floral scent. PM conditioned response did not differ from generalized response to the pear floral scents (PM vs. Pear: Z = − 0.844, P = 0.6759; Fig. 1B), but they did differ from that to apple floral odour (PM vs. Apple: Z = 4.664, P < 0.0001; Pear vs. Apple: Z = 4.357, P < 0.0001; Fig. 1B), showing that the mimic we chose was specific to the pear floral fragrance.

### Similarity of mimic odours to the floral scent

**Figure 2:**
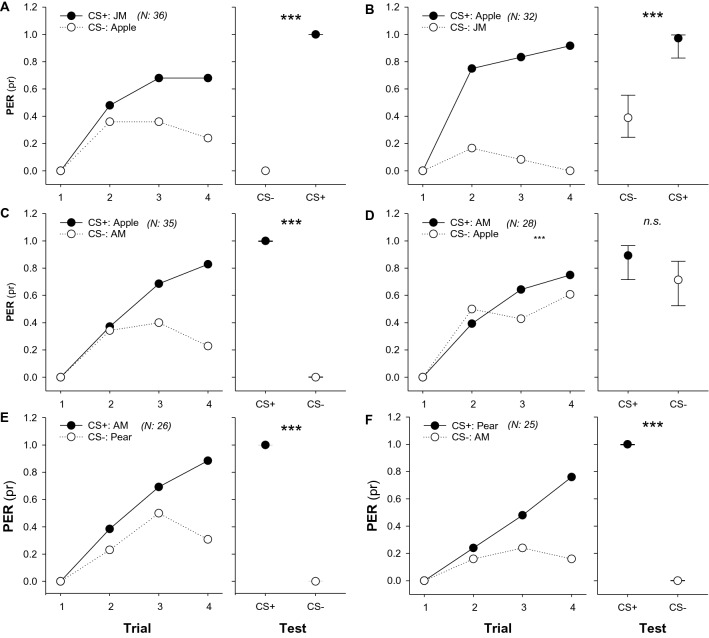
Discrimination between mimic odours and natural floral scents. (**A**,**B**) Discrimination was evaluated towards the single presentation of the apple natural floral odour and the jasmine mimic (JM) (right panels) after a differential proboscis extension reflex (PER) conditioning (left panel), where both apple natural odour and JM were used as rewarded (CS+) and non-rewarded stimulus (CS−). Asterisks indicate significant differences between tested odours (***, p < 0.001). (**C**,**D**) Differential PER conditioning between the apple natural floral odour and the apple mimic (PM), where both odours were used as rewarded (CS+) and non-rewarded stimulus (CS−). No difference (n.s.) at test indicates that bees could not discriminate between AM and the unrewarded apple natural scent (**D**). (**E**,**F**) Differential PER conditioning between the pear natural floral odour and the apple mimic (AM), where both odours were used as rewarded (CS+) and non-rewarded stimulus (CS−). Asterisks indicate significant differences between tested odours (***, p < 0.001) showing that bees could discriminate between AM and the pear natural scent. The experimental subjects were all foraging bees naïve for the CSs that had no access to any pear or apple tree. Numbers between brackets indicate sample size. Circles indicate the proportion of PER and bars (in test) show the 95% confidence intervals. Package ‘emmeans’, version 1.8.0. https://github.com/rvlenth/emmeans.

PER paradigm enables to train bees to discriminate between two odours if one is paired with a sucrose reward (rewarded conditioned stimulus, CS+) and the second is presented as non-rewarded conditioned stimulus, CS−^34^. Following this procedure, we assess to what extent the bees were able to discriminate the synthetic mimics from their natural flower scents. Conditionings were performed using the synthetic mixtures PM and AM and the natural apple or pear floral scent, either as CS+ and CS−. To confirm that bees were able to discriminate between a synthetic mixture and a natural floral blend, we conditioned a first group of bees using a jasmine synthetic mimic (JM) and the apple floral scent as both rewarded (CS+) and non-rewarded stimulus (CS−) and observed that bees could discriminate them (CS+: JM vs. CS−: Apple; Z = − 13,511.965, P < 0.0001; Fig. 2A; CS+: Apple vs. CS−: JM; Z = − 3.744, P = 0.0002; Fig. 2B). Bees could also discriminate the apple natural blend as CS+ and the AM as CS− (CS+: Apple vs. CS−: AM; Z = − 4.905, P < 0.0001; Fig. 2C), but could not discriminate these odours in the opposite situation (CS+: AM vs. CS: Apple; Z = − 1.626, P = 0.1040; Fig. 2D). Furthermore, bees could also discriminate between AM and the pear floral scent with both odours as CS+ and CS− (CS+: AM vs. CS−: Pear; Z = − 3.744, P = 0.0002; Fig. 2E; CS+: Pear vs. CS−: AM; Z = − 13,511.965, P < 0.0001; Fig. 2F). Although discrimination between AM and apple natural scent was not symmetric, results indicate that AM might be functional in conditioning bees toward the apple floral odour. A similar non symmetrical response during differential conditioning was observed when bees were trained to discriminate between PM and pear natural scent (Supplementary Fig. S1). Then, we chose the PM and AM, whose conditioned responses generalized and could not be discriminated from either the pear or the apple natural blends (Fig. 2 and Supplementary Fig. S1) to scent the sucrose solution we offered into the hives that would pollinate the apple and pear trees.

### Colony activity by feeding mimic-scented food in apple and pear crops

**Figure 3:**
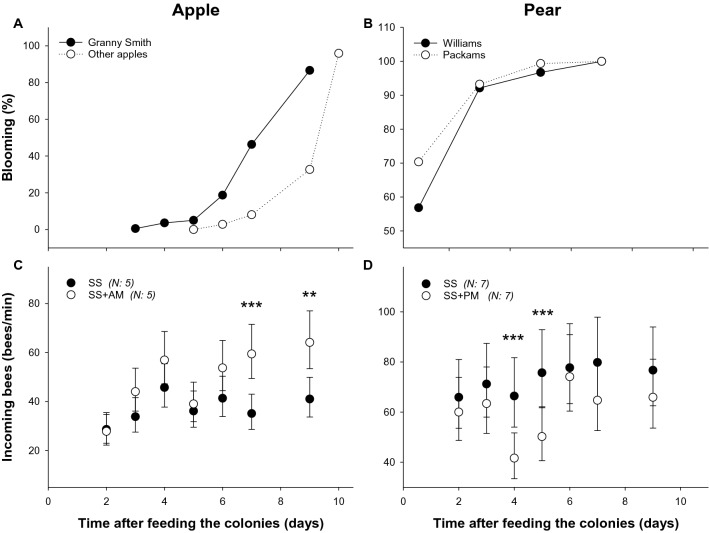
Effect of the mimic odours on the activity of colonies that pollinate apple or pear trees. Percentage of blooming of the dominant variety of apple tree (**A**) or pear trees (**B**) during the experimental period. Colonies were fed either unscented sucrose solution (SS) or apple mimic-scented sucrose solution (SS + AM) (**C**) or pear mimic-scented sucrose solution (SS + PM) (**D**). Incoming bees were calculated as the counts after the offering of the solutions (2, 3, 4, 5, 6, 7 and 9 days). Asterisks indicate significant differences between the specified treatments (***, p < 0.001; **, p < 0.01). Circles indicate the mean values and bars show the 95% confidence intervals. Numbers between brackets indicate the number of colonies monitored per treatment. Package ‘glmmTMB’ version 1.1.4. https://github.com/glmmTMB/glmmTMB.

The offering of PM and AM-scented sucrose solution was used as a standardised procedure to establish olfactory memories expected to guide foragers to the target crops. We showed that colonies fed AM-scented sucrose solution (SS + AM) increased their nest entrance activity compared to unscented sucrose solution (SS) treated colonies (treatment: df = 68, Chi^2^ = 141.7, P = 0.0008). The nest entrance activity of the colonies, explored by means of the bees incoming rate, increased more in SS + AM than SS colonies three days after the offering of scented sucrose solution, a trend that became significant from the seventh day of the experiment (day 2: Z = 0.180, P = 0.8573; day 3: Z = − 1.748, P = 0.0805: day 4: Z = − 1.550, P = 0.1210; day 5: Z = − 1.824, P = 0.0682; day 7: Z = − 3.634, P = 0.0003; day 9: Z = − 3.162, P = 0.0016; Fig. 3C), when flowering of ‘Granny Smith’ apple trees reached 65% of open buds and other apple varieties exceeded 15% (Fig. 3A). Bees incoming rates before treatment were considered in the analysis as covariate. On the contrary, the rate of incoming bees of colonies settled in a pear plot was higher in colonies fed SS than in colonies fed SS + PM (treatment: df = 96, Chi^2^ = 126.75, P = 0.0009). Such differences became significant on day 4 and 5 of the experiment (day 4: Z = 3.054, P = 0.0023; day 5: Z = 2.728, P = 0.0064; Fig. 3D), when almost all pear trees of both dominant varieties (‘Packham’ and ‘Williams’) were in bloom (Fig. 3B).

**Figure 4:**
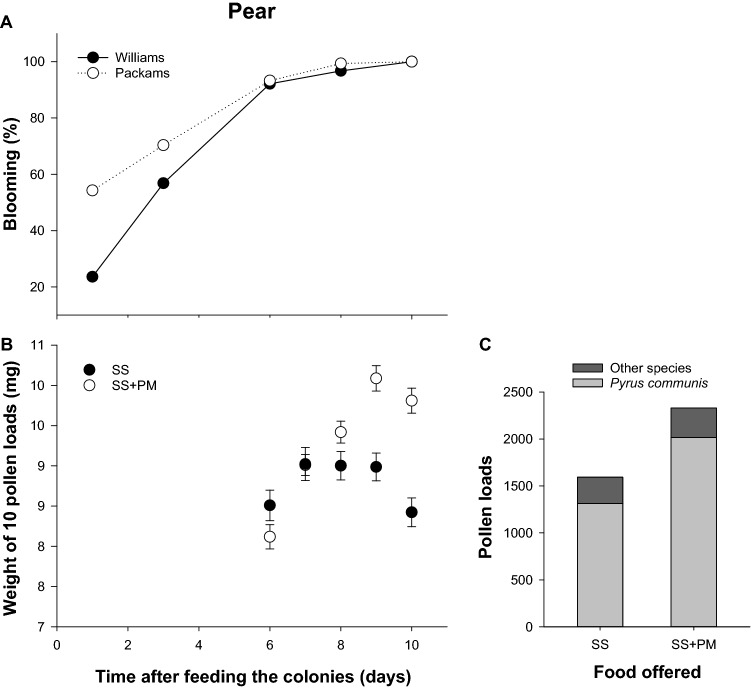
Effect of the pear mimic odour on the pollen foraging. (**A**) Percentage of blooming of the dominant variety of pear tree during the experimental period. As an example is shown one colony fed unscented sucrose solution (SS) and one colony pear mimic-scented sucrose solution (SS + PM). (**B**) Size of pear pollen loads trapped at the colony entrance is expressed in mg of 10 pollen loads along the experimental period. (**C**) Number of pollen loads accumulated during days 3, 7 and 8 after the offering of pear mimic-scented sucrose solution (SS + PM) or unscented sucrose solution (SS) for the same colonies. Pollen traps were placed in one colony treated of each treatment, the samples obtained were classified according to their colour into the categories: *Pyrus communis* (light grey bar) and pollen from other species (dark grey bar).

To assess the possibility that the PM-treatment has a specific effect on pollen collection, the most exploited resource by bees in pear flowers^31^, we quantified the weight and the amount of pear pollen loads (10 corbiculate loads) sampled in traps at the entrance of the hives. The weight of pear pollen loads increased as the level of flowering approached the asymptote in both SS and SS + PM treated colonies (Fig. 4A). However, values of the SS + PM-treated colony turned heavier than those obtained in the SS-fed colony during the last days of the study (8-, 9- and 10-days post treatment; Fig. 4B). Similarly, the amount of pollen loads was about 53.7% higher in the PM-treated colony than in the control one SS (Fig. 4C). Because these observations involved only two colonies, we did not perform statistical analysis.

### Crop yield

**Figure 5:**
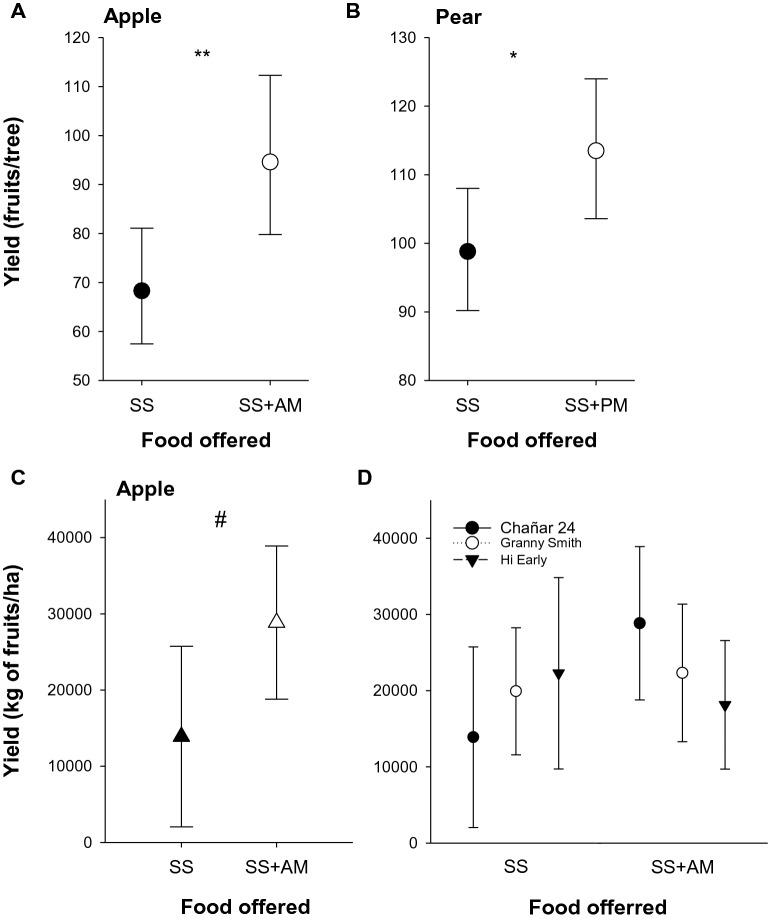
Effect of the offering of mimic-scented food on fruit yield and on plantation yield. (**A**,**B**) Yield was calculated as the counts of fruits per trees in a pair of apple plots and in a pair of pear plots, where 30 trees were surveyed in each. (**A**) Colonies that pollinate each apple plot were fed apple mimic-scented sucrose solution (SS + AM) or unscented sucrose solution (SS). (**B**) Colonies that pollinate each pear plot were fed pear mimic-scented sucrose solution (SS + PM) or unscented sucrose solution (SS). (**C**,**D**). Yield was obtained either from 11 apple plots (varieties: Hi Early, Granny Smith and Chañar 28) that settle 130 colonies in total that had been fed apple mimic-scented sucrose solution (SS + AM) and from 11 apple plots (varieties: Hi Early, Granny Smith and Chañar 28) with 139 colonies that had been fed unscented sucrose solution (SS). Yields are presented for all apple varieties together (**C**) or separately (**D**) Asterisks indicate significant differences between the treatments (*, p < 0.05; **, p < 0.01; ^#^, p = 0.06). Circles indicate the mean values and bars show the 95% confidence intervals. Package ‘ml’, version^60^. Package ‘emmeans’, version 1.8.0. https://github.com/rvlenth/emmeans.

Here, we evaluated apple and pear fruit yields in plots pollinated by honey bees from colonies fed SS + PM/AM or SS. The number of fruits per tree, counted in 30 trees per plot, revealed a 38.5% higher crop yield in the apple plot pollinated by SS + AM-fed colonies than by SS-fed colonies (SS vs. SS + AM: Z = − 2.641, P = 0.0083; Fig. 5A). Similarly, the yield expressed in the number of fruits per tree set of the pear plot pollinated by colonies fed SS + PM was 14.9% higher than the yield of the pear plot that housed colonies fed SS (SS vs. SS + PM: Z = –2.103, P = 0.0355; Fig. 5B). To further investigate the effect of AM on plant productivity, we measured the weight of fruits in terms of kg per surface unit (ha) in plots with different apple varieties (‘Hi Early’, ‘Granny Smith’ and ‘Chañar 24’). We observed that the offering of SS + AM impacts on apple yield (Fig. 5C), although the differences between the two treatments were not significant (treatment: t = 1.978, P = 0.0648). The analysis did not detect differences between varieties (apple variety: F = 0.0294, P = 0.9710), although it can be observed that ‘Granny Smith’ and ‘Chañar 24’ produced more kg of fruits per hectare when pollinated by SS + AM-treated colonies (Fig. 5D). It is worth mentioning that some apple plots presented trees without flowers, through which a correction factor has been considered at the time to evaluate the yield data (Supplementary Table S4).

## Discussion

Apples and pears are the most important crops in the global pome fruit market which makes improving their pollination a major challenge. Here, we encompassed different experiments supporting that the offering of mimic-scented sucrose solution can be used as a tool to improve the efficiency of pollination services provided by honey bees in apple and pear trees. Based on mechanisms that enable social learning, we conditioned honey bees by giving them precocious access to olfactory information that predict the oncoming food sources, with effects on colony activity and crop yield. Our results confirm previous findings on the use of scented food to bias bee foraging preferences to a monocrop such as sunflower^26^ and extend its relevance to crops that occur in heterogeneous environments and face constraints in accessing bee pollination services. Our findings highlight the importance of olfactory information experienced inside the hive for the selection of food sources within complex agricultural setting.

From the chemical ecology perspective, there is consensus that insects do not need to assess all the single odorant components to recognize a bouquet^35^. On the contrary, it seems that insects’ olfactory system has evolved to process complex stimuli just by a small subset of odours. Furthermore, generalisation enables animals to respond in the same way to stimuli that are different but similar^36,37^. This is essential for the foraging behaviour of bees, as they can respond to olfactory cues that characterise the same type of flower, even if they do not present the same volatile emissions^38^. Based on the bees’ perception and generalisation ability, learning a simple mixture that mimicked the floral aroma of apple or pear was enough to bias the bees’ foraging preferences.

Bees constantly need to adjust foraging responses according to fluctuations in the availability and profitability of food sources^39^. Inside the nest, gustatory and/or olfactory information of nectar can be incidentally transferred to, and learned by, colony mates when the resource is unloaded and/or shared via trophallaxis^20,40^. It has been reported that honey bees, as the dominant visitors of apple flowers, reduce their visits to the orchard once an alternative mass flowering crop, like the oilseed rape blooms in the surrounding^29^. However, to what extent a mass flowering crop can impact pollination services of other crop that flower simultaneously but offers a complementary reward is less well known. Our results show that differences in the type and quality of resources available in the environment interact with the olfactory information provided inside the nest, to bias bees´ foraging preferences.

We observed that colonies (controls) located in apple orchards reached an increase in their level of nest entrance activity at the very beginning of the flowering period. However, the nest entrance activity of colonies treated with SS + AM continued increasing until apple bloom was almost complete. The activity at the nest entrance of AM-treated colonies would be due to an increased nectar foraging activity on the apple flower^31,33^. Previous studies showed about 80% of the foragers that visit apple flowers collect nectar, which is abundant and concentrated in terms of sugars^31^. Because the proportion of nectar foragers tends to outnumber pollen foragers, an increase in the number of foragers turning to apple nectar collection could explain the increase we observed in the nest entrance activity.

On the other hand, pear flowers offer much pollen but little nectar. In pear crops we observed that the activity of PM-treated colonies decreases but the amount of pollen pear flowers increased about 50% compared to the controls. It is expected that this experimental procedure of feeding with scented sucrose solution mainly activates nectar foragers, as the in-hive olfactory memories have been established using sucrose solution as reinforcement. However, new evidence suggests that nectar and pollen foraging are not independent processes and that environmental conditions motivate bees to switch between nectar and pollen foraging. In this sense, nectar foragers that encounter sources of decreasing quality show a higher probability of transitioning to pollen collection^41^. While we expect memories established with PM + SS food to bias foragers toward pear flowers (searching for a high-quality nectar source), the low quality of the nectar encountered^31^, might induce bees to switch to pollen collection, which is abundant in the pear flowers. In addition, we could expect that nectar foragers that do not switch to pollen collection would become unemployed after the encounter with nectar of poor quality, leading to a lower activity of nectar forager.

Differences in activity between treated and control colonies become significant not immediately after the offering of food but several days later, suggesting that bees needed to adjust their learned responses based on updated information of the apple and pear flowers. Mimic-memories may have biased the foraging preferences of some bees directly to the target. Moreover, bees that experienced the mimics might have been able to acquire the natural scent of the apple flower faster than unexperienced bees. In the apple crop, therefore, the baseline circulation of apple nectar within the nest at the beginning of the flowering period may have had a stronger effect on the acquisition of apple floral cues among bees from AM-treated colonies than control colonies. In addition, AM-experienced bees may have been more attracted by (and receptive to decode the information of) dancers which, returned to the hive imbibed with the apple flower fragrance^22,26,42,43^, thus assisting recruitment and promoting more visits to the apple flower. This may also be the case in PM-treated colonies, where precocious collection of pear pollen might enable information about pear resources to circulate earlier and in a greater extent within the nest than in control colonies, increasing the probability of selective recruitment to the source in question^44^.

The increase in the amount of pear pollen collected suggests that olfactory information learned with sugar as reward is also functional for guiding bees to sources that are mainly visited for pollen. The present results also suggest that pollen foragers might learn differently how to handle pear flowers and improve in this way their foraging efficiency depending on the colony’s treatment. As it has already been reported^31,45^, we observed that the amount of pear pollen collected per foraging bout increased along the blooming period of pear flowers, but this variable achieved higher values for the SS + PM-treated colony than the control one. This observation suggests that learning is involved in the motor skills required to extract pollen^45^.

Regarding to the apple crops, it should be noted that an increase in the density of nectar foragers would not necessarily result in an increase of apple pollination. For several cultivars, honey bees act as nectar robbers by visiting flowers from the side and without contacting the stamens or the stigma^47–49^. However, the increase in the crop yield for some varieties of apple suggests that this procedure can help to increase pollination efficiency in some apple cultivars.

Additionally, the beehives used for pollination service tents to be very active in the vicinity of the nests and their activity is significantly reduced at greater distances^50^. However, we do not rule out some overlapping flying areas among the colonies used in this study. This situation would promote a lack of spatial independence regarding the honey bee foraging range, which depends on the landscape, and had been reported from 500 m to 5.5 km from the honey bees^52–53^. Additionally, the present experimental design not optimized for controlling the resource limitation effect of orchard^54,55^. Despite these issues, these preliminary results show that the orchard surrounding where the AM/PM-treated colonies were located show increased yields. Further experiments are needed to confirm these results.

Bee pollination has overwhelming effect on yield^2,6,7,10^. Compared to hand and self-pollination, it increased fruit size, seed set, germination rates and fruit quality^10^. However, aspects related to insect behaviour in general, and cognition abilities in particular, have been underestimated as critical processes underlying yield in pollinator-dependent crops. In our study the increasing general activity of AM-treated colonies and the higher amount of pear pollen collected of PM-treated colony positively correlates with crop yield estimated by fruits per tree. On the other hand, when we extended yield measurements, calculated as weight of fruits per unit area) to apple plantations of different varieties, which were in different environments and landscapes, we found an increase, though not significant, in crop yield in plots with different apple varieties pollinated by colonies fed with AM-scented sucrose solution, suggesting that the mimic may be a useful tool but not generalizes equally to all apple varieties. As a summary, our results show that the offering of mimic scented food is a tool that promotes precision pollination to help improving yields in pome crops.

## Materials and methods

### Study sites and colonies

All the experiments were carried out during the apple and pear blooming seasons of 2007, 2008, 2011, 2013 and 2014 in different locations of the province of Rio Negro, Argentina, while some laboratory experiments performed in the city of Buenos Aires. We used individual foragers of *Apis mellifera* L. and their colonies containing a mated queen, brood, and food reserves in ten-frame Langstroth hives. All beehives used had similar sizes and the same management history from the beekeeper. The honey bees studied belonged to commercial Langstroth-type hives rented to pollinate these plots. Each hive had a fertilized queen, 3 or 4 capped brood frames, reserves and approximately 15,000 individuals^56^.

### Testing generalization of memories from pear mimic odours to pear and apple natural floral scents

The absolute conditioning assays were performed in the laboratories of the School of Exacts and Natural Sciences of the University of Buenos Aires (34° 32′ S, 58° 26′ W), Buenos Aires, Argentina. We used honey bee foragers collected at the entrance of the hives settle in the experimental field of the School of Exacts and Natural Sciences. The apple (‘Granny Smith’ and ‘Red Delicious’ varieties) and pear (‘Packham’ and ‘D’anjou’ varieties) bud samples that we used as conditioned stimuli (CS) during the conditioning were collected at the end of the blossom of 2011 in Ingeniero Huergo (39° 03′ 27.5″ S; 67° 13′ 53.5″ W), province of Río Negro, Argentina, and taken to the laboratory in the city of Buenos Aires, Argentina, to be used within the following 2 days.

We first developed the three different synthetic mixtures (PM, PMI and PMII) that could be generalized to the fragrance of the pear flower by foraging bees. The pear synthetic mixtures were formulated considering the previously reported volatile profile of pear blossoms^57^. Then, we chose the synthetic mixture most perceptually similar to the pear flower fragrance and measured its generalisation response to the apple flower fragrance to test the compounds’ specificity. The chemical compounds used to prepare the different synthetic mixtures for the behavioural assays were obtained from Sigma-Aldrich, Steinheim, Germany. The compounds used for the three pear mixtures (PM, PMI and PMII) were composed by alpha-pinene, 2-ethyl-hexanol, (R)-(+)-limonene, and (±)-linalool. For details of the PM and mixture proportions see Patent PCT/IB2018/055550^58^.

To test generalization, we took advantages of the fact that honey bees reflexively extend their proboscises when sugar solution is applied to their antennae^59^. The proboscis extension reflex (PER) can be used to condition bees to an odour if a neutral olfactory stimulus (CS) is paired with a sucrose reward as unconditioned stimulus, US^60^. Conditioned honey bees extend their proboscises towards the odour alone, a response that indicates that this stimulus has been learned and predicts the oncoming food reward. Conditioned bees can generalize such a learned response to a novel odour if it is perceived like the conditioned one (CS). Then we performed three absolute PER conditionings where we paired each of the three PMs with a sucrose-water solution (30%) reward along three learning trials (exp. 4.2a). Afterwards, pear floral scent was presented as novel odour to test generalization. Based on the generalization level to the pear odour, we chose the synthetic mixture that showed the highest generalisation towards pear flower fragrance, and we used it in all the experiments that follow. In an additional 3-trial PER conditioning with the chosen mixture, we quantified generalisation towards both the pear and apple fragrances as novel stimuli (exp. 4.2b).

The experimental bees were all foragers, captured from colonies that had no access to any pear and/or apple tree, hence completely naïve for the CSs. Immediately after capture, bees were anaesthetized at 4 °C and harnessed in metal tubes so that they could only move their mouthparts and antennae^60^. They were fed 30% weight/weight unscented sucrose solution for about three seconds and kept in a dark incubator (30 °C, 55% relative humidity) for about two hours. Only those bees that showed the unconditioned response (the reflexive extension of the proboscis after applying a 30% w/w sucrose solution to the antennae) and did not respond to the mechanical air flow stimulus were used. Trials lasted 46 s and presented three steps: 20 s of clean air, 6 s of odour presentation (CS) and the last 20 s of clean air. During rewarded trials (CS), the reward (US, a drop of 30% w/w sucrose solution) was delivered upon the last 3 s of CS presentation. The synthetic mixtures (PM) were delivered in a constant air flow (15 ml/s) that passed through a 1 ml syringe containing 4 µl of the synthetic mixture on a small strip of filter paper. On the other hand, pear and apple floral volatiles were swept from a 100 g of fresh pear buds (var. ‘D’Anjou’ and ‘Packham’) or apple buds (var. ‘Granny Smith’, ‘Gala’ and ‘Red Delicious’) inside a kitasato by means of an air flow (54 ml/s).

### Testing discrimination between mimics and natural floral scents

The differential conditioning assays were performed in a field laboratory in Ingeniero Huergo, province of Río Negro, Argentina. Conditioning trials with AM as CS were carried out in September 2007 and 2008, prior to the beginning of flowering of the fruit trees. Conditioning trials with PM as CS were carried out in September 2011 in the same area (Ingeniero Huergo, province of Río Negro, Argentina). Apple and pear bud samples used as CS were collected in plots that start blooming located around Ingeniero Huergo, but distant (more than 1 km) from the plot where we collected the bees. The bud samples presented the following varieties: *M. domesticus* sp., ‘Granny Smith’, ‘Gala’, and ‘Red Delicious’; *P. communis* sp., ‘Packham’ and ‘D’Anjou’.

With the aim to develop a synthetic mixture that presents difficult to discriminate with the fragrance of the apple flower by foraging bees, an apple synthetic mixture (AM) was formulated considering the previously reported volatile profile of apple blossoms^61^. The chemical compounds used to prepare the apple synthetic mixtures for the behavioural assays were obtained from Sigma-Aldrich, Steinheim, Germany. Apple mimic (AM) was composed by benzaldehyde, limonene and citral. For details of the AM proportions see Patent AR20110102441^62^. Jasmine mimic (JM) was a commercial extract obtained from Firmenich S.A.I.C. y F, Argentina.

If the synthetic mixture chosen were perceptually similar to the apple flower fragrance, experimental bees should have difficult to discriminate to the apple flower fragrance to test the compounds’ specificity. Thus, we performed differential PER conditioning between synthetic mixtures (AM and Jasmine mimic, JM) or between synthetic mixtures (AM or JM) and the apple natural fragrance. We followed a differential PER conditioning^34^ to assess to what extent the bees were able to discriminate the synthetic mimics from their natural flower scents. PER differential conditioning consisted of four pairs of trials, four rewarded trials (CS+) and four non-rewarded trials (CS−) that were presented in a pseudo-randomized manner. Conditionings were performed using the synthetic mixtures PM and AM and the natural floral scents, pear and apple, either as CS+ and CS−. We followed the same procedure that in 3.3 to capture the bees and to present the stimuli during trials.

### Feeding protocol

We used the offering of scented sucrose solution in the hive as a standardized procedure to establish long-term olfactory memory in honey bees^23–24,26,63^. Scented sucrose solution was obtained by diluting 50 µl of PM or AM per litre of sucrose solution (50% weight/weight, henceforth: w/w). For the ‘apple’ series, colonies were fed 1500 ml of sugar solution offered in an internal plastic feeder for 2 days, about 3 days before the apple trees began to bloom. For the ‘pear’ series, hives were fed 500 ml of sugar solution that we spread over the top of the central frames. Both feeding procedures have been found to be functional for establishing olfactory in-hive memories^26^. Depending on the pear varieties, the scented sucrose solution was offered when the pear trees were 10–40% in bloom.

### Colony activity

The effects of the AM-treatment on colony nest entrance activity were studied in 18 colonies located in an agricultural setting of apple and pear trees in Ingeniero Huergo, on an 8-ha plot, half of which was planted with apple trees (varieties: ‘Granny Smith’, ‘Gala’ and ‘Red Delicious’) and the other 4 ha with pear trees (varieties: ‘Packham’ and ‘D’anjou’). The effect of the PM-treatment on colony activity was studied in 14 colonies located in three adjoining pear plots (total surface: 8 ha) in Otto Krause (39° 06′ 22″ S 66° 59′ 46″ O, Supplementary Fig. S5), province of Río Negro, Argentina. The varieties of these plots corresponded to ‘Packham’ and ‘Williams’. Pollen collection (exp. 4.5.2) was also studied in colonies located in these plots.

We focused on the nest entrance activity since once the first successful foragers return to the hive and display dances and/or unload the food collected, it promotes the activation or reactivation of inactive foragers and, in a minor proportion, those hive mates ready to initiate foraging tasks^39,65–67^. Then, we choose number of incoming bees as an indicator of colony foraging activity, since most of these bees are expected to return from foraging sites^33^. Thus, we compared the activity level at the nest entrance between 7 SS + PM-treated colonies and 7 SS-treated colonies. We also compared the nest entrance activity level between 5 colonies treated with SS + AM and 5 colonies fed with SS. This activity value was estimated by the number of incoming foragers at the entrance of the hive for one minute, every morning at the same time (10:30 a.m.) during the entire experiment (9 consecutive days). A first measurement was done one day before feeding the colonies (used as covariate) and 7 measurements afterwards.

We measured the amount of pollen loads collected by two colonies: one fed with SS + PM and one fed with SS. Pollen loads were collected using conventional pollen traps (frontal-entrance trap), consisting of a wooden structure with a removable metal mesh inside. Pollen samples were collected for 3 days, two hours per day during the late morning, 3, 7 and 8 days after the offering of SS + PM or SS. Pollen pellets identified based on pollen colour as coming from the pear flower or from other species were separated and counted. In addition, we estimated the weight of pear pollen loads during a 5 days period, from 6 to 10 days after the offering of scented or unscented sucrose solution. To reduce measurement error, pollen loads were weighed in groups of 10.

### Crop yield

Pear crop yield was studied in pear plots in General Roca (39° 02′ 00″ S; 67° 35′ 00″ O, Supplementary Fig. S4, Supplementary Table S3), province of Río Negro, Argentina. In an area of 15.2 ha (4 plots of 3.8 ha each), 45 beehives were equidistantly located in groups. We measured the number of fruits per tree set of 30 trees in the surrounding areas of the PM-treated colonies (2 groups of 8 hives) and control colonies (2 groups of 8 hives). A third group category contained 13 untreated colonies. The varieties of the pear trees were ‘D’Anjou’ and ‘Packham’.

Apple crop yield estimated by means of number of fruits per plant was studied in General Roca (Supplementary Fig. S2, Supplementary Table S1), province of Río Negro, Argentina. We measured fruit set in the two plots that covered a surface of 3.8 ha and contained a total of 74 colonies distributed in groups (the control plot, 39 SS-treated-colonies treated with SS; and the treated plot, 35 SS + AM-treated-colonies treated with SS + AM). The varieties of the apple trees were ‘Red Delicious’ (clone 1), ‘Royal Gala’ and ‘Granny Smith’.

A second studied on apple fruit yield by means of kg of fruits per hectare was performed in Coronel Belisle (39° 11′ 00″ S 65° 59′ 00″ O, Supplementary Fig. S3, Supplementary Table S2), province of Río Negro, Argentina. Four apple plots with ‘Granny Smith’, ‘Hi Early’ and ‘Red Delicious’, clone 1 varieties of 15.4 ha each were randomly assigned to different treatments (treated plot 1, 40 SS + AM-treated-hives treated with SS + AM; treated plot 2, 40 SS + AM-treated-hives treated with SS + AM; control plot 1, 40 SS-treated-hives treated with SS; control plot 2, 40 SS-treated-hives treated with SS).

During the fruit harvest, the fruit yield was estimated in the surroundings (150 m around) of two groups of 8 colonies each. We fed one group SS + PM and the other unscented sucrose solution (SS). Yield was estimated as the number of fruits per trees in 30 randomly selected trees within each area, alternating the counts between the North and South faces of the plots. Following the same procedure, we also estimated the number of fruits per trees in the surroundings of two groups of 14 colonies each that pollinated apple crops. Again, we fed one group SS + AM and the other SS. Additionally, a total of 218 colonies in General Roca and 180 colonies in Coronel Belisle have been separated in the two experimental groups, in which yield had been provided by the producer and expressed in kg of fruits per ha. It is worth remarking that in some plots the distance between treated and control beehive groups was around 300 m, suggesting that might have been overlapping flying areas between treated and control hives. Additionally, the apple fields studied in the surrounding of Coronel Belisle, presented many trees without flowers. It was considered that the absence of flowers in numerous trees would bias the counts performed in those fields. Then, to quantify this situation, which might be associated with the masting phenomenon^68^, samples with the proportions of trees without flowers for every 20 trees in each plot was done. Trees that had between 80 and 100% of their surface devoid of flowers were considered “without flowers” trees, and “trees with available flowers” those that had more than 20% of their surface covered with flowers. An average of 30% of the trees within these plots were devoid of flowers. Thus, a correction factor was considered to evaluate the yield data provided by the grower per plot analysed (Supplementary Table S4).

### Statistics

All statistical analyses were performed with R Core Team 2019^69^. For Experiment 4.2 and 4.3, we analysed PER proportion by means of a binomial multiplicative generalized linear mixed model using the “glmer” function of the ‘lme4’ package^70^.

For experiment 4.2a we considered the pear mimics (three-level factor corresponding to PM, PMI and PMII) and the event (two-level factor corresponding to 3rd trial and test) as fixed factors and each “bee” as a random factor.

For experiment 4.2b we considered the tested odours (three-level factor corresponding to Apple, Pear and PM) as fixed factors.

For experiment 4.3 we considered the tested odours (two-level factor corresponding to CS+ and CS−) as fixed factors. Post hoc contrasts were conducted on models to assess effects and significance between fixed factors using the “emmeans” function of the ‘emmeans’ package version 1.7.0^71^ with a significance level of 0.05.

For experiment 4.5.1 we analysed “rate of incoming bees” using a generalized linear mixed model. As Poisson model for incoming bees was overdispersed^72^, we used a negative binomial distribution using the ‘glmmTMB’ package (function ‘glmmTMB’^73^. We considered “treatment” [two-level factor corresponding to SS + AM (or SS + PM) and SS], “days” (7-level factor corresponding to the date after treatment), the rate of incoming bees before the offering of food (to control for pre-existing colony differences) as covariate (a quantitative fixed effects variable), and “colony” as a random factor.

For experiment 4.6, we analysed fruits per trees by means of a negative binomial multiplicative generalized linear mixed model using the “log” function of the ‘ml’ package^70^. Post hoc contrasts were conducted on models to assess effects and significance between fixed factors using the “emmeans” function of the ‘emmeans’ package version 1.8.0^71^ with a significance level of 0.05. For experiment 4.6b we analysed “yield” (as weight of fruits per unit area) using a general linear mixed model. We checked homoscedasticity and normality assumptions (Levene and Shapiro–Wilk tests, respectively). We considered “treatment” (two-level factor corresponding to SS + AM and SS) and “apple varieties” (3-level factor corresponding to *Hi Early, Granny Smith* and *Chañar 28*) as fixed factors and “location” (2-level factor corresponding to General Roca and Coronel Belisle) as random factors.

## Supplementary Information

Supplementary Information.

## Data Availability

The datasets generated for this study are available upon request to the corresponding author.
