# Supplementary material for: In-hive learning of specific mimic odours as a tool to enhance honey bee foraging and pollination activities in pear and apple crops
Source: Sci Rep. 2022 Nov 28;12:20510. doi: 10.1038/s41598-022-22985-5 (PMC9705528; doi:10.1038/s41598-022-22985-5)
Supplement: Supplementary file 1 — Supplementary Information. [file 41598_2022_22985_MOESM1_ESM.docx]

Supplementary Material

**In-hive learning of specific mimic odours as a tool to enhance honey bee foraging and pollination activities in pear and apple crops**

Walter M. Farina^1,2,#^*, Andrés Arenas^1,2,#^, Paula C. Díaz^1,2,+,^^, Cinthia Susic Martin^1,2,+^, and María J. Corriale^3,4^

*^1^Laboratorio de Insectos Sociales, Departamento de Biodiversidad y Biología Experimental, Facultad de Ciencias Exactas y Naturales, Universidad de Buenos Aires, Buenos Aires, Argentina.*

*^2^Instituto de Fisiología, Biología Molecular y Neurociencias (IFIBYNE), CONICET-**Universidad de Buenos Aires, Buenos Aires, Argentina.*

*^3^Grupo de Estudios sobre Biodiversidad en Agroecosistemas, Departamento de Ecología, Genética y Evolución, Facultad de Ciencias Exactas y Naturales, Buenos Aires, Argentina.*

*^4^Instituto de Ecología, Genética y Evolución de Buenos Aires (IEGEBA), CONICET-Universidad de Buenos Aires, Buenos Aires, Argentina.*

*^^^Current address: Instituto Nacional de Medicina Tropical, Administración Nacional de Laboratorios e Institutos de Salud (ANLIS), Ministerio de Salud de la Nación, Puerto Iguazú, Misiones, Argentina.*

*Corresponding author: Email: [walter@fbmc.fcen.uba.ar](mailto:walter@fbmc.fcen.uba.ar)

**Figure S1**

**Fig. S1. Discrimination between pear mimic odour and the natural floral scent.** Discrimination was evaluated towards the single presentation of the pear natural odour and the pear mimic (PM) at the test (right panel) after a differential proboscis extension reflex (PER) conditioning (left panel), where both odours were used as rewarded (CS+) and non-rewarded stimulus (CS-). **A)** Pear natural odour (floral natural scent) was used as CS- and the pear mimic (PM) as CS+. No difference (n.s.) at test indicates that bees could not discriminate between PM and the unrewarded pear natural scent. **B)** Pear natural odour (natural floral scent) was used as CS+ and PM as CS-. Asterisks indicate significant differences between tested odours (***, p<0.001). The experimental subjects were all foraging bees completely naïve for the conditioned odours that had no access to any pear tree. Numbers between brackets indicate sample size. Circles indicate the proportion of PER and bars (in test) show the 95% confidence intervals. Package ‘emmeans’, version 1.8.0. https://github.com/rvlenth/emmeans.

**Table S1.** Plots of apple trees in the crop field of General Roca (province of Rio Negro, Argentina) evaluated in this study. It is detailed the planted varieties of ‘Red Delicious’ and their pollinator varieties. Number of tree files are also presented.

| **Plot** | **Red Delicious Clone 1** | **Red Delicious Clone 2** | **Pollinator variety** | **Planted files** |
| --- | --- | --- | --- | --- |
| **4** | Chañar 28 |  | Granny Smith | 93:00:31 |
| **5** |  | Chañar 34 | Royal Gala | 00:100:33 |
| **8** | Chañar 28 | Chañar 34 | Royal Gala | 01:73:25 |

**Table S2.** Plots of apple trees in the crop field of Coronel Belisle (province of Rio Negro, Argentina) evaluated in this study. It is detailed the planted varieties of ‘Red Delicious’ and their pollinator varieties. Number of tree files are also presented.

| **Plots** | **Red Delicious Clone 1** | **Red Delicious Clone 2** | **Pollinator variety** | **Planted files** |
| --- | --- | --- | --- | --- |
| **16 a, b, c, d** | Hi Early |  | Granny Smith | 74:74 |
| **17 a, b, c, d** | Hi Early |  | Granny Smith | 92:92 |
| **30 a, b, c*, d** | Chañar 28 |  | Granny Smith | 100:100 aprox |
| **31 a, b, c, d** | Chañar 28 | Hi Early | Granny Smith | 100:100:100 aprox |

*Due to a marked difference in the composition of the planted varieties, plot 30c was excluded from the study.

**Table S3.** Plots of pear trees in the crop field of General Roca (province of Rio Negro, Argentina) evaluated in this study. It is detailed the planted varieties and their number of tree files.

| **Plot** | **Var. 1** | **Var. 2** | **Files Var1: Var2** |
| --- | --- | --- | --- |
| **6** | D´Anjou | Packam´s | 50:48 |
| **7^*^** | D´Anjou | Packam´s | 20:50 |
| **6** | D´Anjou | Packam´s | 50:48 |
| **12** | D´Anjou | Packam´s | 21:77 |
| **11** |  | Packam´s | Only 1 variety |

*This plot has planted 28 files of the Williams variety.

**Table S4.** Plots of the Coronel Belisle apple fields with a correction factor (masting) applied to the yield values obtained. This factor is multiplied by the yield value obtained in the corresponding plot.

| Varieties / plants / treatment | Plot | Masting |
| --- | --- | --- |
|  |  |  |
|  |  |  |
| Granny Smith: Hi Early 171:1291 SS + AM | 16 a | 0.8 |
|  | 16 b | 0.85 |
|  | 16 c | 0.5 |
|  | 16 c | 0.65 |
| Granny Smith: Hi Early 203:1603 SS | 17 a | 0.95 |
|  | 17 b | 0.95 |
|  | 17 c | 0.65 |
|  | 17 c | 0.65 |

| Granny Smith: Chañar 28 259:2198 SS | 30 a | 0.15 |
| --- | --- | --- |
|  | 30 b | 0.45 |
|  | 30 d | 0.6 |
| Granny Smith: Chañar 28: Hi Early 344:1358:1427 SS + AM | 31 a | 1 |
|  | 31 b | 0.7 |
|  | 31 c | 0,85 |
|  | 31 d | 0,7 |

**Figure S2**


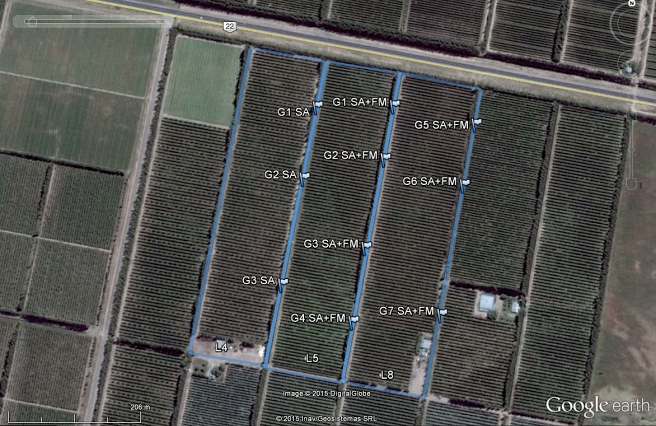

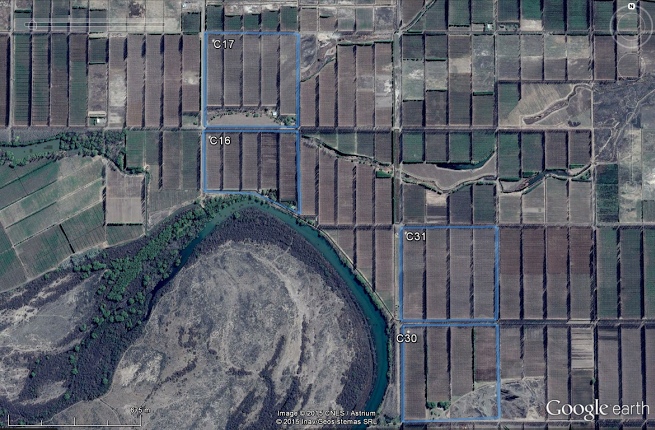


**Fig. S2.** Plots of apple crops managed in a field near General Roca, province of Rio Negro, Argentina (top figure). Relative locations studied in an apple field in Coronel Belisle, province of Rio Negro, Argentina (bottom figure). L= plot. C=Frame. Boxes indicate plots with apple trees (L4, L5, L8, above; C16, C17, C30, C31, below). G= groups of beehives. They are numbered consecutively for each treatment in each plot. SA: unscented sucrose solution treated beehives; SA + FM: AM-scented sucrose solution treated beehives. (Map data: Google, CNES / Astrium, INAV, version 3. <https://github.com/iNavFlight/inav>).

**Figure S3**


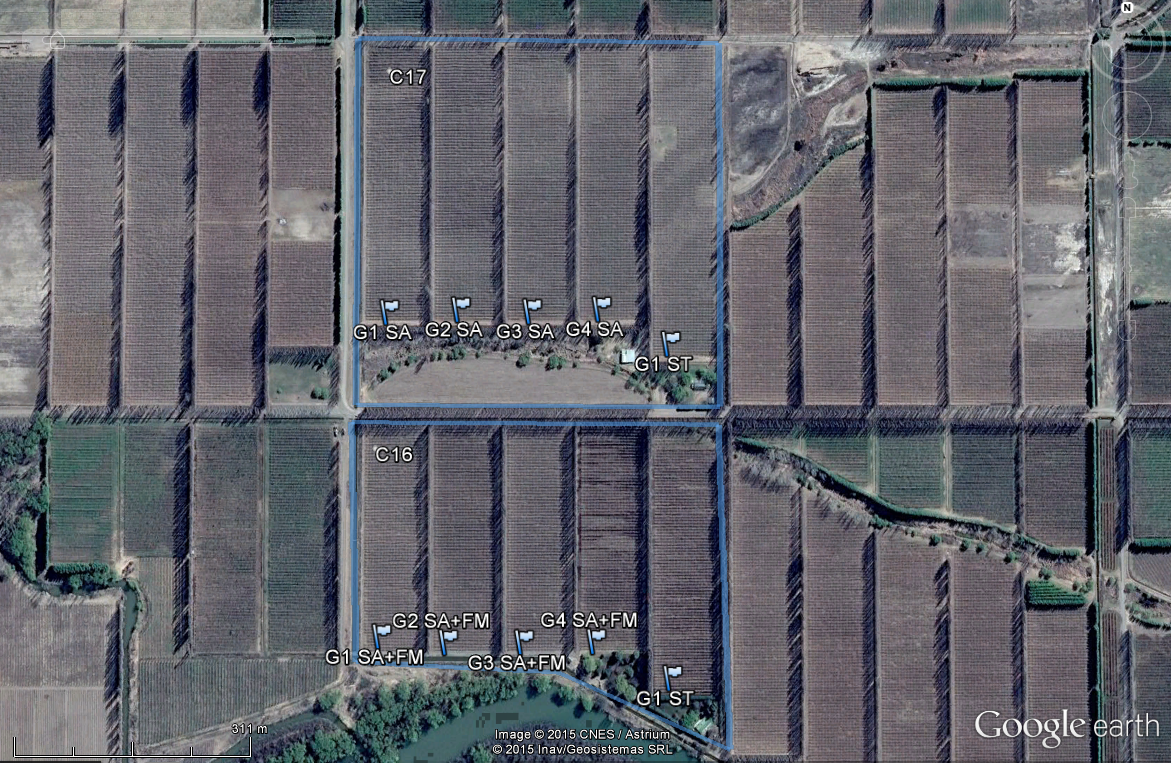

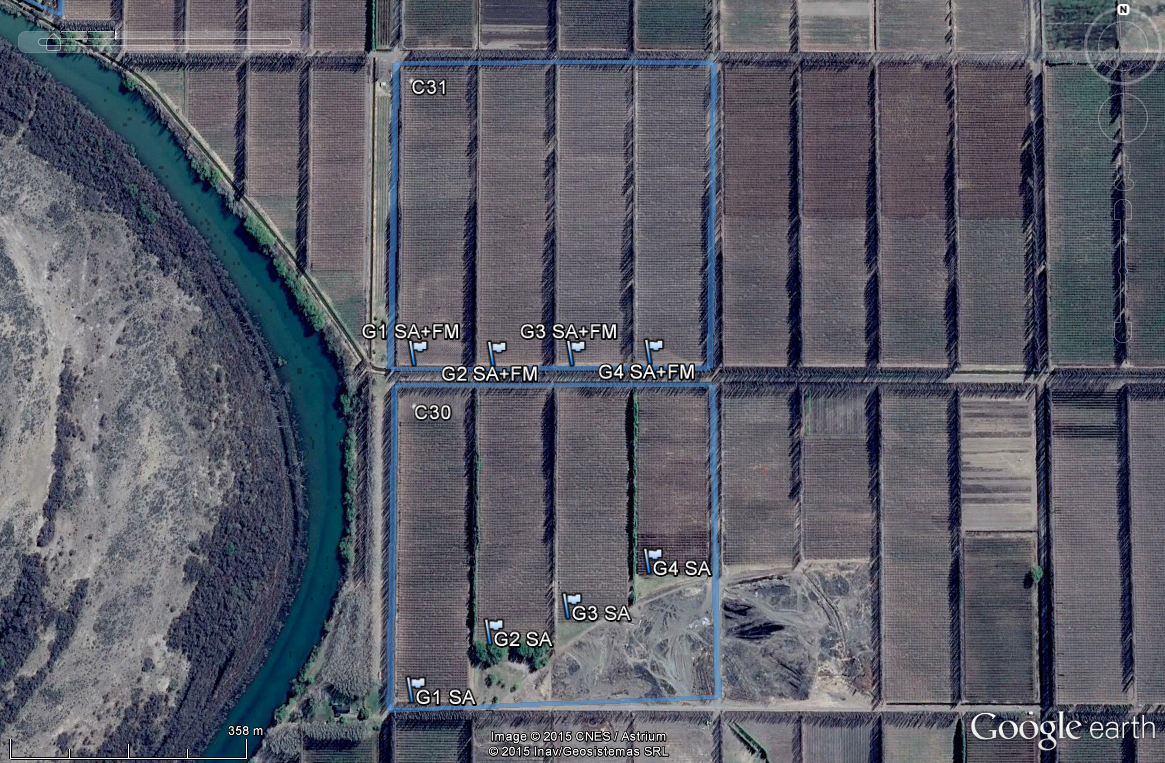


**Fig. S3.** Detail of the apple plots studied in the Coronel Belisle field. Frames C16 and C17 consist of 5 plots each, in which each plot has been identified with the letters *a* to *e*, from left to right of the frame (top figure). Frames C30 and C31 consist of 4 plots each, in which each plot has been identified with the letters *a* to *d*, from left to right of the frame (bottom figure). G= groups of beehives. They are numbered consecutively for each treatment in each lot. SA: unscented sucrose solution treated beehives; SA + FM: AM-scented sucrose solution treated beehives; ST: untreated beehives. (Map data: Google, CNES / Astrium, INAV, version 3. <https://github.com/iNavFlight/inav>.

**Figure S4**


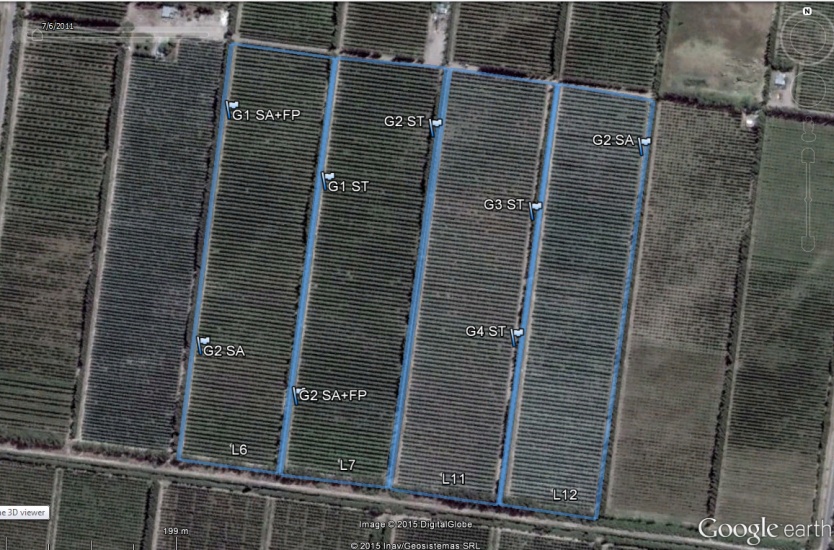


**Fig. S4.** Plots of pear crops managed in a field near General Roca, province of Rio Negro, Argentina. Boxes indicate plots with pear trees: L6, L7, L11 and L12. G= groups of beehives. They are numbered consecutively for each treatment in each plot. SA: unscented sucrose solution treated beehives; SA + FP: PM-scented sucrose solution treated beehives; ST: untreated beehives. (Map data: Google, Digital Globe, INAV, version 3. <https://github.com/iNavFlight/inav>).

**Figure S5**


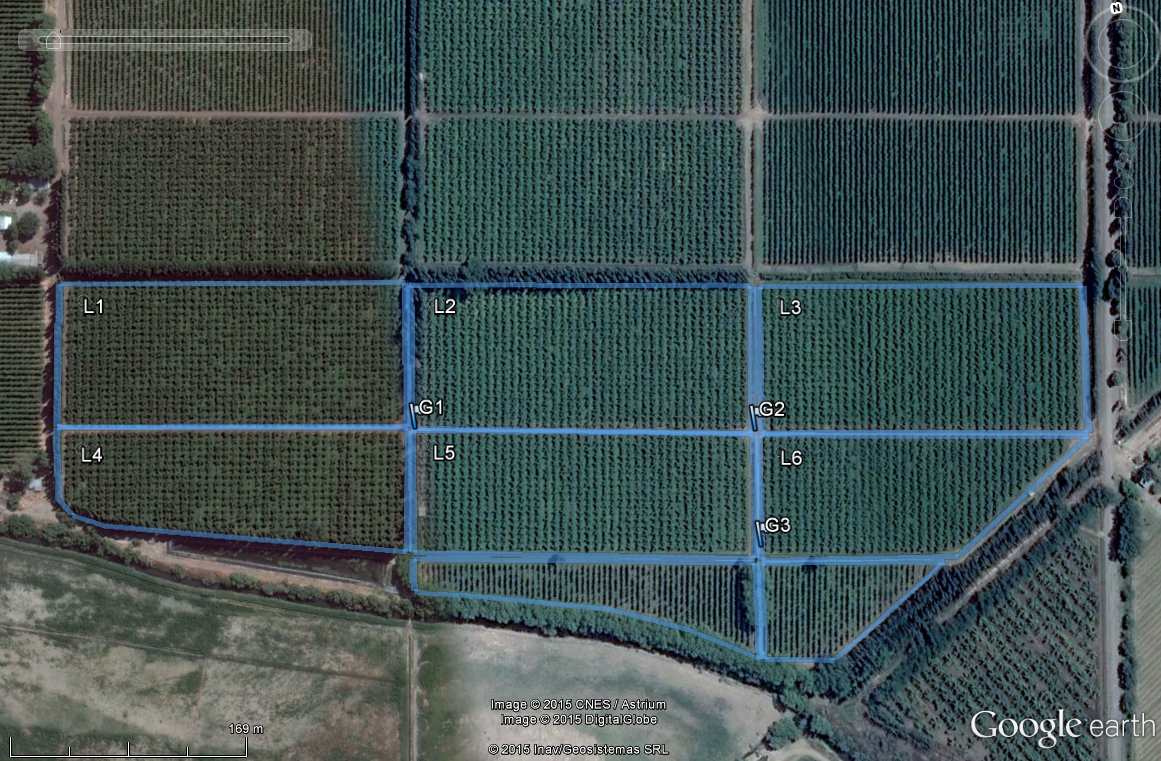


**Fig. S5.** Plots of pear crops managed in a field near Otto Krause, province of Rio Negro, Argentina. L= plot. Boxes indicate lots with pear trees (L1-L6). G= groups of beehives. The three groups add 32 hives and among them 14 were randomly selected for this study. (Map data: Google, CNES / Astrium, Digital Globe, INAV, version 3. <https://github.com/iNavFlight/inav>).
